# Nanomaterial-Based Electrochemical Immunosensors for Clinically Significant Biomarkers

**DOI:** 10.3390/ma7064669

**Published:** 2014-06-19

**Authors:** Niina J. Ronkainen, Stanley L. Okon

**Affiliations:** 1 Department of Chemistry and Biochemistry, Benedictine University, 5700 College Road, Lisle, IL 60532, USA; 2 Department of Psychiatry, Advocate Lutheran General Hospital, 8South, 1775 West Dempster Street, Park Ridge, IL 60068, USA; E-Mail: Stanley.Okon@advocatehealth.com; 3 Formerly of the Department of Pathology, University of Illinois at Chicago, MC 847, 840 S. Wood St., Suite 130 CSN, Chicago, IL 60612, USA

**Keywords:** biomarkers, biosensors, cancer diagnostic tools, carbon nanotubes, conducting polymers, electrochemical detection, metal nanoparticles, graphene, immunosensors, nanowires, psychiatry, quantum dots

## Abstract

Nanotechnology has played a crucial role in the development of biosensors over the past decade. The development, testing, optimization, and validation of new biosensors has become a highly interdisciplinary effort involving experts in chemistry, biology, physics, engineering, and medicine. The sensitivity, the specificity and the reproducibility of biosensors have improved tremendously as a result of incorporating nanomaterials in their design. In general, nanomaterials-based electrochemical immunosensors amplify the sensitivity by facilitating greater loading of the larger sensing surface with biorecognition molecules as well as improving the electrochemical properties of the transducer. The most common types of nanomaterials and their properties will be described. In addition, the utilization of nanomaterials in immunosensors for biomarker detection will be discussed since these biosensors have enormous potential for a myriad of clinical uses. Electrochemical immunosensors provide a specific and simple analytical alternative as evidenced by their brief analysis times, inexpensive instrumentation, lower assay cost as well as good portability and amenability to miniaturization. The role nanomaterials play in biosensors, their ability to improve detection capabilities in low concentration analytes yielding clinically useful data and their impact on other biosensor performance properties will be discussed. Finally, the most common types of electroanalytical detection methods will be briefly touched upon.

## 1. Introduction

Improvements in the early detection and monitoring of certain diseases have resulted in a shift from treatment-based medicine towards preventive medicine. There has been a dramatic increase in cancer rates during the last few decades, especially in developed countries, and cancer has become the leading cause of death for many age groups. Early detection and diagnosis of certain types of cancer, for example, has become possible through the use of biomarkers such as CA125 and highly sensitive biosensor devices and assays. Early detection is often the key to successful treatment and patient survival. Diagnosing cancer based on the patient’s presenting symptoms can lead to a delay in initiating treatment given that symptoms usually appear when tumors are large or have metastasized. Therefore, noninvasive, sensitive, and accurate screening methods for the early detection of cancer are critical and remain an active area of research.

Biomarker detection has also made the development of personalized treatment plans for certain diseases possible. The treatment plan can take into consideration factors such as a patient’s gender, age, height, weight, diet, and environment. It has been predicted that the use of biomarkers in the detection and treatment of a wide range of diseases will continue to grow in the future [1]. Since biomarkers have emerged as key players in preventive medicine, the need for accurate, reproducible, efficient, and easy detection and quantification by non-specialists has become of the outmost importance. Affordable detectors, fast sample processing times, minimal labor, small sample volumes, and ability to detect multiple biomarkers simultaneously are also very important. Furthermore, the storage stability and resistance to degradation of reagents in biosensors are important factors to consider. Also, as with any analysis method, automated or semiautomated, easy to use devices are preferred. Small, mass produced, and relatively inexpensive enzyme-based electrochemical biosensors have been used by public and medical personnel, for example, in the management of diabetes by glucose monitoring as well as in the measurement of lactate and cholesterol for decades. However, biosensors which detect biomarkers of clinical significance such as tumor markers or hormones are relatively new analytical tools in medicine.

Biosensors are devices that register a biological change or reaction which is converted into a signal that can be detected and quantified [2]. A typical biosensor contains biological recognition molecules that are highly selective and specific for the analyte(s) of interest. In electrochemical biosensors, the biological recognition molecules bind with a particular analyte on or near an electrochemically active interface that may incorporate nanomaterials giving rise to a measurable signal. The electric transducer or the detector device that is in contact with the electrochemically active interface, usually an electrode, converts that biochemical reaction into an electrical signal that may be amplified further by a signal processor. The signal processor includes computer software that converts the electrical signal into a form that may be displayed onto a computer screen. Common transducer types in biosensors include; optical, thermal, magnetic, and acoustic. Since 2000, there have been many scientific articles that have been published showing a significant improvement in the quality of signals originating from biochemical reactions (*i.e.*, higher signal to noise ratios) in electrochemical biosensors that incorporate nanomaterials [3,4,5,6,7,8].

A major reason why immunosensors and immunoassays are so popular in clinical analysis is the characteristic and exceptionally high selectivity, sensitivity and specificity that an antibody exhibits for its target antigen [2]. For this reason, particle-based immunomagnetic assays have been popular in biomarker detection for some time. Moreover, in recent years, the incorporation of nanomaterials into biosensors and assays has further improved their limits of detection and sensitivity. The remarkable sensitivity of nanomaterial-based biosensors and assays also provides opportunities for detecting infectious organisms and biothreat agents at concentrations that cannot be measured by current and conventional methods. However, use of biosensors in detecting disease biomarkers will be the primary focus of this article.

## 2. Nano- and Biomaterials

### 2.1. Nanomaterials

Nanotechnology and advances in microfabrication technology have played a critical role in the development of biosensors. Nanoscale materials have been incorporated into various enzymatic biosensors, genosensors, and immunosensors. The overall trend to make smaller and more portable devices with better performance has lead to incorporating nanomaterials into biosensors. These nanomaterials include carbon nanotubes (CNTs), graphene, nanowires, magnetic nanoparticles, and quantum dots (QDs). The word “nano” which was derived from the Latin word *nanus*, “dwarf”, has become commonplace in the context of miniaturization. The term nano typically refers to anything with at least one dimension in the size ranging from 10^−7^ m to 10^−9^ m. Nanomaterials can be biological, organic, or inorganic. Most nanomaterials tend to be very stable, and the variety of nanoparticles and nanoscale molecules makes multiplexing within a biosensor possible. Table 1 gives examples of various types of nanomaterials and their relative sizes. Biological nanomaterials that are responsible for recognizing and interacting with the analyte of interest (the biomarker) include macromolecules such as antibodies and DNA as well as artificial, synthetic molecular recognition elements that can be customized for each application such as aptamers [2]. Organic and inorganic nanomaterials such as nanoparticles or nanowires are often used in biosensors to amplify the binding event by using some measurable change in a property such as electrical conductivity of a nanowire or change in the way the nanoparticle complex interacts with light. These nanomaterials are often used to modify or interact with the transducer which is responsible for measuring and transmitting the signal generated in the presence of the target analyte; the biomarker. In electrochemical biosensors, the use of nanomaterials helps to increase charge and electron transfer. In addition, nanomaterial-modified sensing surfaces have improved electrochemical properties as a result of low background current and higher signal-to-noise ratios [9]. The nanoparticles themselves may play the role of electrochemical labels or can be used as a vehicle to host hundreds or thousands of electroactive labels or biological capture molecules. Although gold nanoparticles are the most studied of inorganic nanoparticles, particles of silver (Ag), cobalt (Co), copper (Cu), iron (Fe), platinum (Pt), palladium (Pd), iridium (Ir), nickel (Ni) and others as well as mixtures of these metals have also been incorporated into biosensors [10,11,12,13,14,15,16,17,18,19,20,21,22,23]. Metal oxides have also been used in biosensors [23,24,25]. Gold (Au) nanoparticles are widely used in enzyme-based biosensors such as enzyme electrodes due to their extraordinary catalytic activity [26]. Organic nanomaterials contain various allotropes of carbon such as carbon powder, graphene, carbon nanowires, carbon capsules, carbon nanotubes (CNTs), and carbon nanotube arrays [26]. Table 1 gives examples of these types of nanomaterials and their typical sizes. Many hybrid nanomaterials such as graphene-Pt nanoparticles [27] and Pt nanoclusters embedded in polypyrrole nanowires [28] have also been studied.

**Table 1:** Common nano- and biomaterials.

Type	Examples	Type of molecule/species	Typical diameters ^a^
Biological	Antibodies	Y-shaped proteins	10–15 nm
	Aptamers	artificial, single stranded polynucleotides	3–5 nm
	DNA	polynucleotides	3–50 nm
	Enzymes	globular proteins	6–40 nm
	Cancer cells	cells	10,000–40,000 nm
Inorganic	Metal nanoparticles	Ag, Au, Co, Cu, Fe, Ir, Ni, Pd	1–100 nm
	Semicoductors	quantum dots	2–20 nm
	Nanowires	SiO_2_, TiO_2_, Si, GaN, InP, In_2_O_3_, Au, Cu, Pt, Ni	1–50 nm
Organic	Carbon nanospheres and capsules	poly(divinyl)benzene, polyaniline, polypyrrole, polyacetylene	10 nm–1 μm
	Carbon nanotubes (CNTs)	hollow carbon cylinders	–
	–	SWCNT	1–2 nm
	–	MWCNT	2–50 nm
	Graphene	sp^2^ hybridized carbon sheets with hexagonal honeycomb-shaped lattices	–

^a^ These ranges of diameters are estimates based on assorted data in the literature.

Nanomaterials are comprised of thousands of molecules or atoms which are aggregated into shapes such as rods, wires, tubes, pores, particles, and arrays of these shapes. The chemical and physical properties of nanoscale materials are significantly different from those in bulk matter. Many of these differences are the result of much larger surface-area-to-volume ratios which can result in chemical reactions being magnified in nanostructures. For example, the large surface area of nanomaterials such as CNTs permits high loading of biocatalysts or biological capture molecules responsible for analyte recognition and ultimately improves quantification of the reaction that is being analyzed. Nanomaterials are popular in the design and fabrication of biosensors utilizing electrochemical detection due to their stability, electrical conductivity, and various favorable structural and catalytic properties [26]. In many biosensors, the electrode surface is used as the solid support onto which nanomaterials, antibodies or antigens are immobilized. In addition to traditional electrode surfaces, nanomaterials have been used to modify screen-printed electrodes that are common in many biosensor designs. The choice of the immobilization procedure or method of placing the nanomaterials for the biorecognition of molecules on the electrode surface or the solid support is a very critical step in the biosensor development. However, these procedures and methods are beyond the scope of this review and are described in more detail along with their associated challenges and advantages in the various recent manuscripts, theses, and books [19,25,29,30,31].

In addition to the emergence of various nanomaterial-based biosensors, manufactured nanomaterials have been incorporated into a variety of commercial products such as sun screens, cosmetics, medical delivery devices, Band-Aids, detergents, disinfectants, pesticides, and construction materials since the United States federal government first promoted the National Nanotechnology Initiative in 2000 [32]. More recently, the National Nanotechnology Initiative (NNI) Strategic Plan which was released in February 2014 [33] outlined the vision, goals, and objectives for coordinated research and development initiatives in the discovery, development and deployment of nanoscale science and technology in the United States. Three out of the four primary goals in the NNI Strategic Plan are highly relevant to the scope of this manuscript. They are:

(1) To advance world-class nanotechnology research and development;
(2) To foster the transfer of new technologies into products for commercial and public benefit;
(3) To develop and sustain educational resources, a skilled workforce and the supporting infrastructure and tools to advance nanotechnology.

Due to the increase in manufacturing of nanomaterials resulting from the rise in their popularity in various commercial products and scientific applications, the need for assessing their environmental safety and potential impact on human and animal health have also become active areas of research [34]. As they represent a relatively new class of materials, their fate, transport, exposure, and toxicity upon introduction into the environment must be carefully studied. Unlike bulk matter and molecular species, nanoparticles which are nanometer-scaled colloidal particles tend to be carried along by the surrounding medium such as air or water as they do not settle under normal gravitational conditions [35]. They also behave like small colloidal particles in that they do not diffuse to any significant extent [35]. Some of the nanomaterials also undergo chemical reactions once released into the environment with changes in the metal species and their charges. Physical processes such as abrasion can also be a problem with some nanomaterials. The release of nanomaterials such as copper and silver nanoparticles (CuNPs and AgNPs respectively), carbon nanotubes (CNTs), and nanoscale cerium oxide (Nanoceria) into the environment is studied by the US Environmental Protection Agency (EPA), other agencies, and environmental laboratories.

In addition to the environmental safety concerns, several limitations prevent the widespread use of nanomaterials in mass-produced biosensors. The surface modification protocols used to prepare the biosensors incorporating nanomaterials tend to be long and sometimes complicated [36]. Furthermore, nanomaterials tend to be quite costly if purchased from specialized companies and may not be available in large quantities. Some nanomaterials are even not commercially available.

Nanomaterials have been introduced to various types of signal transduction technologies in biosensors. Electrochemical biosensors can be divided into two main categories based on the type of biological recognition process related to the target analyte, *i.e.*; biocatalytic devices such as enzyme-based electrodes and affinity sensors such as immunosensors and nucleic acid based sensors [2]. New methods for immobilization of biorecognition components (typically antibodies, nucleic acids, cells, bacteria or enzymes) onto organic or inorganic nanomaterials and other surfaces have also significantly improved the detection capabilities of these biosensors. However, in this manuscript, the primary focus will be on immunosensors and immunoassays that utilize electrochemical detection (with antibodies being the biorecognition component of the biosensor) for certain biomarker analytes. Combining bioselectivity and specificity of antibodies with the numerous and advantageous chemical and physical properties of nanomaterials and the well-known advantages of electrochemical detection has allowed the development of biosensor devices with significantly improved performance. We will investigate how advanced nanomaterials are utilized in a subset of biosensors called immunosensors, what role they play in the sensor device or the assay, how they improve the detection capabilities, and describe some examples of electrochemical immunosensors and immunoassays for biomarker detection. We will also discuss the basic science of the most common electrochemical detection techniques utilized in immunosensors in Section 5. We will begin by describing the most common types of biological nanomaterials or nanostructures that are utilized in clinical applications.

### 2.2. Biological Nanomaterials

#### 2.2.1. Antibodies as the Biorecognition Element

The immunoaffinity reaction in the immunosensor often involves an irreversible binding of the antigen (Ag) to the antibody’s (Ab) binding site(s). A high degree of complementarity is necessary between the Ab’s binding site (a paratope) and the compatible binding region of the Ag (the epitope) in order for the noncovalent interactions to result in the formation of a stable Ab-Ag complex. This highly specific immunoaffinity reaction is an interaction between the Ab molecule (a large Y-shaped glycoprotein), and the Ag (the biomarker of interest) which is often a protein or a hormone. Each Ab molecule is said to be bivalent as it has two identical Ag binding sites. Most antibodies used in immunosensors for biomarker detection and quantification are monoclonal antibodies (MAbs) and recognize a specific structural feature of the biomarker [37]. MAbs are all identical in their primary protein structure resulting in them being more specific for their corresponding biomarker Ag than polyclonal antibodies (PAbs). Unlike PAbs, MAbs only recognize a small, single chemically unique epitope on the Ag molecule and are able to distinguish between very small chemical differences on the surface of the biomarker. Ultimately, the specificity of the Abs immobilized in the immunosensor varies depending on the nature and quality of the antibodies used in fabricating the immunosensor. In immunosensors, this biochemical reaction resulting in the Ab-Ag complex formation ultimately produces a detectable signal by way of the transducer component in the biosensor. Of note, special care must be taken when selecting the assembly conditions of the Abs with the nanomaterials because different Ab molecules have unalike electrostatic properties therefore adjustments must be made in the Ab-nanomaterial conjugate assembly conditions of different Abs even when the same nanomaterials are used [38].

#### 2.2.2. Enzyme Labels in Immunosensors

Like many immunoassays, some immunosensors utilize enzymes such as horseradish peroxidase (HRP) or alkaline phosphatase (AP) as biochemical labels. The electrochemical signal that is generated depends on the concentration of electroactive product generated by the enzyme-catalyzed reaction which is affected by the amount of enzyme label bound to the immunosensor. The amount of Ag captured by the Abs in the immunosensor, and ultimately the concentration of Ag in the biological sample, are related to the amount of enzyme label present during the detection step. The use of enzyme labels provides significant signal amplification in the immunosensor as each enzyme molecule rapidly generates many electrochemically detectable product molecules, resulting in extremely low detection limits.

One example of an immunosensor application using the enzyme label alkaline phosphatase (AP) is the microfabricated immunosensor for the determination of salivary cortisol in real saliva samples [39]. Microfabricated Au electrodes encased in a microfluidic chamber to form an immunoelectrochemical device were functionalized to immobilize the primary cortisol capture antibodies by EDC/NHS-biotin attachment chemistry. The AP enzyme label attached to the cortisol antigen, via a secondary antibody binding in a sandwich assay format, served as the enzyme label. AP is a commonly used enzyme label which hydrolyzes orthophosphate from a wide variety of phosphate esters under alkaline conditions. During the biochemical reaction, the AP enzyme that is attached to bound cortisol and Abs catalyzes the conversion of redox inactive *p*-nitrophenyl phosphate (*p*NPP) to electrochemically active product *p*-nitrophenol (*p*NP) by cleavage of a phosphate (Equation (1)). The resulting *p*NP was detected at room temperature as an oxidative peak between 0.9 and 1.1 V (*vs*. Ag pseudo-reference electrode) using cyclic voltammetry (CV). After 10 min of incubation, the detection limit of cortisol by CV was 0.27 ng/mL (0.76 nmol/L) at a 50 mV/s scan rate.

*p*NPP + H_2_O → *p*NP + P*_i_* (1)

### 2.3. Types of Nanomaterials and Nanostructures in Biosensors and Assays

#### 2.3.1. Nanowires and Nanowire Arrays

Nanowires are nanostructures, with diameters in the order of nanometers and no restriction on the length. Nanowires as long as one millimeter (10^−3^ m) have been synthesized although a more typical length for a nanowire is about one micrometer (10^−6^ m) [26]. A common width-to-length ratio of a nanowire is 1:1000 or higher, which allows these nanostructures to be viewed as one-dimensional (1-D) [26]. Their very small sizes and 1-D structures result in unique physical properties of nanowires when compared to three-dimensional wires. Nanowire conductance can be carefully controlled by synthesizing the wire from different substrates. Nanowires have been synthesized from metals (Ni, Cu, Au, Pt, *etc.*), metal oxides (ZnO, SnO_2_, Fe_2_O_3_), silicon/indium/gallium semiconductors (Si, InP, GaN) and silicon/titanium oxide insulators (SiO_2_, TiO_2_). Metallic nanowires have the highest conductance, while insulators have the lowest.

#### 2.3.2. Quantum Dots

Quantum dots (QDs) are inorganic semiconductor nanoparticles or nanocrystals with a typical diameter of 2–10 nm [26]. Their electronic properties are in-between those of discrete molecules and of bulk semiconductors [40]. They also exhibit unique electro-chemiluminescent properties, fluoresce intensely, have excellent fluorescence quantum yields, high absorbances, and size-tunable narrow-emission spectra [41]. Due to these properties, quantum dots are an excellent alternative to organic dyes as fluorescent labels in biomolecules such as proteins or oligonucleotides [42]. QDs have great potential as probes for *in vivo* and *in vitro* cancer detection and imaging. Recently, they have also been used in biosensor devices for detecting viruses such as influenza [43,44,45]. QDs have a core made of an inorganic material such as cadmium selenide or telluride (CdSe, CdTe or CdSeTe) surrounded by a shell material such as zinc sulfide (ZnS) and an outside layer of a thin organic polymer film which can be functionalized with primary amines, carboxyl groups, or other groups that allow the direct immobilization of biomolecules or a covalent attachment of biomolecules via a linker molecule. The organic polymer films with functionalized layers help reduce photolytic, oxidative, and mechanical degradation of QDs which could lead to biocompatibility problems and toxicity if free cadmium ions were released [46]. Issues that need further study are the limited knowledge on QD clearance in living systems, problems with reproducibility, and sensitivity to oxidation and photolysis. Once these limitations can be overcome, QDs have great potential in cancer diagnostics due to their sensitivity and multiplexed detection capabilities. Krejcova *et al.* [43] determined and characterized the metal part (Cd) of the quantum dot label (CdTe) used to mark viruses by differential pulse anodic stripping voltammetry (DPASV). Hu *et al.* [47] reported a QD-based microfluidic protein chip for the direct detection of cancer biomarkers in serum.

#### 2.3.3. Carbon Nanotubes and Other Allotropes of Carbon

There has been a significant surge in studies on the fundamental physical and chemical properties of carbon nanomaterials and their applications in the engineering field, the physical sciences (such as electroanalytical chemistry, biotechnology) and nanodiagnostics since the discovery of carbon nanotubes (CNTs) in 1991 [48]. CNTs, which are now used extensively in nanofabrication, were also some of the first nanomaterials along with gold and magnetic nanoparticles that were incorporated into immunosensors and enzymatic biosensors. CNTs are graphene sheets which are wound into a cylindrical shape with diameters of a few nanometers and lengths up to hundreds of microns [25,26]. These hollow cylinders may be left open or closed at either end with caps containing pentagonal carbon rings. All graphene-based nanomaterials, such as CNTs, are made of sp^2^ hybridized carbon, imparting exceptional tensile strength around 50 times greater than steel [49]. Each carbon atom is covalently bound to its 3 adjacent neighbors resulting in a seamless structure with hexagonal honeycomb-shaped lattices [50]. CNTs have been prepared in a variety of structures differing in thickness, length, number of layers, and type of helicity. CNTs also have very high surface-area-to-weight ratios. The electrical properties of CNTs vary greatly depending on the structural differences between CNTs resulting in some CNTs acting like metals, while others act more like semiconductors. The current carrying capacity of certain CNTs can be 1000 times greater than that of copper wire [51]. CNTs can be produced by chemical vapor deposition (CVD) [52,53,54], electric arc discharge (EAD) [55], laser vaporization of a graphite electrode [56] or laser ablation (LA) [57]. Different methods for growing CNTs that are discussed in more detail elsewhere in a review article as well as a reference book- (see references) can be chosen to prepare nanomaterials with different structural forms and corresponding unique physical properties [25,26].

Furthermore, single or multi-wall CNTs can be produced. Both single-wall carbon nanotubes (SWCNT) and multi-wall carbon nanotubes (MWCNT) are now widely used in biosensor applications. MWCNTs have the same general layout as SWCNTs (which are 1–2 nm in diameter), except there are multiple layers of CNTs, each enclosing each other like the rings of a tree trunk with an interlayer distance of 0.34 nm resulting in diameters ranging from 2 to 50 nm [58]. CNTs have unique chemical, thermal, optical, and mechanical properties [50]. For example, CNTs are very stiff and chemically stable [16,59]. CNTs are thermally stable to above 2800 °C under vacuum and are twice as thermally conductive as diamond [60]. CNTs are electrochemically inert much like other carbon-based materials commonly used in electrochemistry (for example, glassy carbon and graphite) [59]. CNTs are often used as intermediates between gold, glassy carbon, or platinum electrodes and electroactive species in biosensors due to their high conductivity and fast electron transfer rates [26]. The electroactive sites on CNTs are located at the ends of the tubes (both in SWCNTs and MWCNTs) as are most structural defects [59,61,62,63]. When CNTs are assembled into a collection of parallel nanoelectrodes, their individual electric signals combine into an enhanced, detectable signal [64].

CNT-modified electrodes in biosensor applications have shown superior electron-transfer reactions in both small biologically relevant molecules and larger biological macromolecules [65,66]. Biofunctionalization of CNTs by using covalently attached linker molecules or direct immobilization of the biomolecules such as antibodies, bestows additional selectivity of detection on the CNT-based biosensors. The three dimensional cylindrical shape and very large surface area of CNTs allow substantial amount of biomolecules to be incorporated into the biosensor [26]. For example, when Wang *et al.* [67] utilized CNTs as labels for electrochemical immunoassays, about 9,600 enzyme molecules were attached to each 1 micron long CNT which led to a very high signal amplification factor culminating in extremely low detection limits. Electrodes modified by CNTs have been demonstrated to have superior chemical and electrochemical stabilities in both aqueous and non-aqueous solutions [68]. However, the use of SWCNTs in certain types of biosensors can be challenging in many ways such as: possessing surfaces too small to interface with large biorecognition components such as cells, being harder to manipulate during sensor fabrication steps, and not easily biofunctionalized [26]. Furthermore, commercial production of CNTs that are defect-free is costly and labor-intensive [59]. It is also quite challenging to grow CNTs of uniform lengths, prevent CNT aggregation, and minimize the nonspecific binding of proteins to CNT walls [59]. Despite the foregoing limitations and the fact that the electrochemical properties of the two types of CNTs discussed are not yet fully understood, they remain potential candidates for use in amperometric biosensor devices for biomarker detection. Indeed, measurable and significant changes in the electronic behavior of SWCNTs have been reported upon their interaction with various proteins and other biologically relevant molecules [69,70,71,72,73].

Graphene is another nanomaterial that is an allotrope of carbon which is commonly used in biosensors and was discovered in 2004 [74]. It is a two-dimensional, one-atom thick sheet made of pure sp^2^-hybridized carbon in a densely packed crystal lattice with a hexagonal pattern similar to graphite. The high surface area of graphene sheets provides a large number of redox-active sites. Since graphene has the same basic structure as graphite and CNTs, it has many of the same chemical and physical properties. It has high thermal conductivity, high mechanical strength, is biocompatible, and has fast electron transport properties [75]. In addition, graphene is inexpensive, environmentally friendly and highly elastic [76]. Furthermore, it is also easier to immobilize proteins on flat graphene sheets rather than cylindrical CNTs. As is the case with CNTs, the electron transfer between graphene and relevant redox active species occurs primarily at the edges of the graphene sheet and/or at any defects found in the basal plane [76].

#### 2.3.4. Metal Nanoparticles

##### Gold Nanoparticles (GNPs)

Nano-sized colloidal gold (Au) nanoparticles are perhaps the most studied nanomaterial and are still popular in biosensor applications due to their extraordinary catalytic activity. GNPs are manufactured from small octahedral units called primary units. The size and morphology of the nanoparticles can be adjusted, based on the synthesis method employed allowing scientists to optimize the biorecognition molecules’ microenvironment on the electrode surface [25,77]. Like carbon-based nanomaterials, when gold nanoparticles are incorporated into biosensor devices the amount of biological recognition molecules such as antibodies loaded onto the sensor increases significantly [25]. Due to their nano-scale size, GNPs have high surface area to volume ratios providing more available sites for the biological recognition molecules and the analyte. Although gold is an inert metal, the high surface area to volume ratio, surface properties [12,16] and quantum-scale dimensions [78] significantly enhance the catalytic activity of GNPs resulting in increased and measurable electrical signals.

GNPs are typically stored in an aqueous solution, are highly biocompatible and have lower cellular toxicities compared to many other types of nanomaterials used in biomedical applications [79]. As with other nanomaterials, GNPs can be used to modify the surface of the electrode in biosensors based on electrochemical detection. GNPs may be deposited or attached onto the electrode transducer, where they provide an efficient and three-dimensional loading-platform with large surface area for immobilizing biomolecules such as proteins. Furthermore, GNPs help improve electron transfer between the biomolecule and the electrode.

In recent years, GNPs have often been combined with other biocompatible materials in order to prepare hybrids, such as Au-silicon oxide (SiO_2_) hybrids [80], Au-carbon nanosphere hybrids [81], and Au-layered calcium carbonate (CaCO_3_) hybrids [82]. As a result, synergistic properties of the components making up the hybrids, such as improved biocompatibility and stability, have been observed.

##### Other Metal Nanoparticles

Nanoparticles of copper, palladium, cobalt, silver, platinum, and others have also been incorporated into various biosensors [83,84,85,86,87]. For example, Baioni *et al.* [83] prepared 30 nm copper hexacyanoferrate nanoparticles that were immobilized onto fluorine-doped tin oxide electrodes by using the electrostatic deposition layer-by-layer technique resulting in electroactive films with electrocatalytic properties towards hydrogen peroxide (H_2_O_2_) reduction. After immobilizing the glucose oxidase enzyme, the nanoparticle containing film was used as a mediator in a glucose biosensor for detecting the oxidation of glucose to gluconolactone. On the other hand, Li *et al.* [84] fabricated an amperometric glucose biosensor based on an n-alkylamine-stabilized palladium nanoparticles-glucose oxidase modified glassy carbon electrode. The performance of the biosensor was characterized by cyclic voltammetry prior to amperometric measurement of glucose in pH 7.0 phosphate buffer solution and in human blood serum samples.

Metal nanoparticles have been used in various biosensor designs. Of note, Salimi *et al.* [85] electrodeposited cobalt oxide nanomaterials onto a glassy carbon electrode followed by the immobilization of cholesterol oxidase to prepare a sensitive cholesterol biosensor. The cholesterol was quantified using voltammetry and flow injection analysis. Similarly, Liu *et al.* [86] fabricated a H_2_O_2_ biosensor with amperometric detection based on the direct electrochemistry and electrocatalysis of myoglobin that was immobilized on silver nanoparticles-doped carbon nanotubes film using hybrid sol–gel techniques. Meanwhile, Hrapovic *et al.* [87] prepared platinum nanoparticles (diameter = 2–3 nm) and used them in combination with single-wall carbon nanotubes for fabricating glassy carbon or carbon fiber microelectrode-based electrochemical sensors for determination of hydrogen peroxide.

The differences in the morphologies of the metal nanoparticles affect their properties and suitability for use in biosensors. For example, platinum (Pt) nanoparticles with different morphologies such as hollow *versus* solid Pt nanospheres display different electrochemical characteristics when used as labels in immunosensors and in other applications [88]. The hollow nanospheres have been shown to have better catalytic properties, lower density, higher specific surface area, and lower cost due to utilizing smaller amounts of material during fabrication [89].

#### 2.3.5. Nano-Structured Conducting Polymers and Nanocomposites

Conducting polymers (a.k.a. organic conjugated polymers) such as polyacetylene, polyparaphenylene, polyaniline, and polypyrrole are popular in electrochemical biosensors due to their high electrical conductivity or charge transport properties [90]. Conductive polymers are mostly organic compounds with an extended pi-orbital system, through which electrons can move along the length of the polymer [91,92,93]. The electronic and mechanical properties, such as flexibility of the conducting polymers, can be adjusted based on chemical modeling and synthesis methods. Ideal polymers are thermally and environmentally stable, soluble, easily processed, and highly electrically conductive. The electrical conductivity (S/cm) of these polymers changes over several orders of magnitude in response to changes in their applied potentials, pH and environment [94]. These conducting polymers can be grown electrochemically on the entire electrode surface or a well-defined area of the electrode. Like other nano-scale materials, nanomaterials consisting of conductive polymers have significantly improved signals and overall performance relative to more traditional materials used in biosensors due to their larger exposed surface areas [95].

The biological recognition molecules such as antibodies can be immobilized onto conducting polymers without loss of their binding activity, while making the conducting polymers biocompatible and usable in near neutral aqueous sample environments [90]. Conducting polymers are relatively easy to prepare and have been used in the fabrication of inexpensive, accurate, and fast biosensors for use in the medical diagnostic laboratories. Also, other nanomaterials such as cadmium sulfide (CdS) nanoparticles have been incorporated into conductive polymer films for an even greater improvement in the biosensor’s sensitivity [96].

## 3. Biomarkers as Analytes

Biomarkers can be defined as biologically relevant, disease specific molecules that can be quantified using laboratory techniques or instrumental devices. They act as indicators of current or future disease state and include proteins, protein fragments, signaling molecules, DNA markers and cells [97]. Biomarkers can be divided into three categories including: (1) diagnostic biomarkers which assist in early detection of a disease; (2) prognostic biomarkers, which help assess the malignant potential of tumors; and (3) predictive biomarkers, which can be used to differentiate between various cancers and help in designing therapy plans for the patient [41]. The analytical tools for biomarkers must be capable of operating at the level of differential diagnosis and to be specific enough to not produce numerous false-positive results [98]. The use of biomarkers in cancer diagnosis, staging, and monitoring response to cancer therapy is already a well established diagnostic tool. An ideal biomarker for cancer is a protein or a protein fragment that can be detected very early in the patient’s blood or urine, but not detected in healthy individuals [98]. Although cancer biomarkers are of great interest due to the incidence of cancer increasing dramatically over the past few decades, especially in developed countries, biomarkers are also used in other areas of medicine such as psychiatry and endocrinology. Table 2 lists commonly used biomarkers used in cancer diagnosis/prognosis and psychiatry as well as the biomarker type/category.

**Table 2:** Biomarkers used in cancer diagnosis and prognosis and psychiatry [99,100,101].

Cancer type	Biomarkers	Biomarker type
*Biomarkers in cancer diagnosis and prognosis*		
Bladder	BTA	Bladder Tumor Antigen
	BAT	Mononucleotide marker of microsatellite instability (impaired DNA mismatch repair) in Bladder cancers
	FDP	Fibrin degradation products
	NMP 22	Nuclear Matrix Protein
	HA-Hase	Hyaluronic acid-Hyaluronidase (molecule-enzyme/protein)
	BLCA-4	Nuclear Matrix Protein
	CYFRA 21-1	Cytokeratin 19 intermediate filament fragment
Breast	CA125, CA15-3, CA27.29	Cancer antigens/Mucin-like glycoproteins
	CEA	Glycoprotein/Carcinoembryonic protein
	BRCA1/2	Tumor suppressors
	MUC-1	Glycosylated protein
	NY-BR-1	Differentiation antigen
	ING-1	Tumor suppressor
Colon and pancreatic	CEA	Glycoprotein/carcinoembryonic protein
	CA19-9	Sialylated lacto-*N*-fucopentaose II/Cancer antigen
	CA24-2	Sialylated Lewis carbohydrate
	p53	Nuclear phosphoprotein/Tumor suppressor
Esophagus carcinoma	SCC	Squamous Cell Carcinoma antigen
Gastric carcinoma	CA72-4	Cancer antigen/Mucin-like glycoproteins
	CA19-9	Sialylated lacto-N-fucopentaose II/Cancer antigen
	CEA	Glycoprotein/Carcinoembryonic protein
*Biomarkers in cancer diagnosis and prognosis*		
Leukemia	BCR, ABL, PML, BCL1/2, ETO	Chromosomal abnormalities or mutations caused by an error in cell division following meiosis or mitosis
Liver	AFP	Glycoprotein/Fetal protein/Carcinoembryonic protein
	CEA	Glycoprotein/Carcinoembryonic protein
Lung	NY-ESO-1/ESO-1	Cancer testis antigen
	CEA	Glycoprotein/carcinoembryonic protein
	CA19-9	Sialylated lacto-N-fucopentaose II/cancer antigen
	SCC	Squamous Cell Carcinoma antigen
	CYFRA 21-1	Cytokeratin 19 intermediate filament fragment
	NSE	Glycolytic enzyme
Melanoma	Tyrosinase	Oxidase enzyme
	NY-ESO-1/ESO-1	Cancer testis antigen
Ovarian	CA-125	Cancer antigen
	AFP	Glycoprotein/fetal protein/carcinoembryonic protein
	hCG	Glycoprotein heterodimeric (α and β subunits) hormone
	p53	Nuclear phosphoprotein/Tumor suppressor
	CEA	Glycoprotein/carcinoembryonic protein
Prostate	PSA	Serine protease
	PAP	Enzyme
Solid Tumors	EWS, WT1, ASPL, CHOP, FKHR, PAX3	Chromosomal abnormalities or mutations caused by an error in cell division following meiosis or mitosis
Testicular	AFP	Glycoprotein/fetal protein/carcinoembryonic protein
	β-hCG	β subunit of hCG a Glycoprotein heterodimeric hormone
	CAGE-1	Cancer testis antigen
	ESO-1	Cancer testis antigen
Trophoblastic	SCC	Squamous Cell Carcinoma antigen
	hCG	Glycoprotein heterodimeric (α and β subunits) hormone
**Psychiatric Disorder**	**Biomarkers**	**Biomarker type**
*Biomarkers used in other medical fields such as Psychiatry*		
Psychotic symptoms	Cortisol (↑)	Steroid hormone/Glucocorticoid
Major Depressive Disorder	Cortisol (↑)	–
Post Traumatic Stress Disorder	Cortisol (↓)	–
Schizophrenia	Cortisol (↑)	–
Substance Abuse Disorder	Cortisol (↑)	–
Stress (brief or sustained)	Cortisol (↑)	–
Wilson Disease	Ceruloplasmin (↓)	Copper-binding protein/Oxidase enzyme
Hyperthyroidism can present as:	–	–
Mood disorder	Thyroid-stimulating hormone (TSH) (↓), FT4 (↑)	Glycoprotein hormone
*Biomarkers used in other medical fields such as Psychiatry*		
Psychosis	TSH (↓), FT_4_ (↑)	–
Delirium	TSH (↓), FT_4 _(↑)	–
Hypothyroidism can present as:	–	–
Fatigue	(TSH) (↑), FT4 (↓)	–
Depression	(TSH) (↑), FT4 (↓)	–
Memory impairment	(TSH) (↑), FT4 (↓)	–

### 3.1. Cancer and Tumor Biomarkers

Ideal cancer biomarkers would be proteins or protein fragments that can easily be detected and quantified in the patient’s urine or blood (as their collection is non- or minimally invasive), but are not found in healthy individuals [102]. Biopsies which are commonly used to obtain proteins or protein fragments from tissue are usually invasive, uncomfortable and/or painful for the patients. Numerous immunoassays and immunosensors have been developed for isolation and detection of single tumor markers in human serum. Currently used immunological methods include Enzyme-Linked Immunosorbent Assay (ELISA), immunohistochemistry, immunofluorescence, radioimmunoassay, flow cytometry, Western blot, immune-polymerase chain reaction assay, chemiluminescence assay, mass spectrometric immunoassay, and others. The concentration of the tumor biomarkers that are detected in human serum can sometimes be associated with the stages of tumors [103,104,105]. Certain biomarkers for cancer diagnosis, prognosis, and therapy monitoring such as CA125, CA15-3, CEA, and prostate specific antigen will now be described.

#### 3.1.1. Cancer Antigen 125 (CA125)

CA125 (Mucin 16) is an antigenic determinant that was initially detected using the murine monoclonal antibody OC 125 [106] and is associated with a high molecular weight >200 kiloDaltons (kDa) mucin-like glycoprotein [99]. It was isolated in 1981 by a research team led by Drs. Bast and Knapp [106]. It is a serum biomarker for monitoring and follow-up of ovarian, breast, and uterine cancer patients and prognosticating about a patient’s response to various cancer therapies. However, some patients undergoing chemotherapy may exhibit a false decrease in CA 125 levels and therefore it does not always rule out tumor recurrence. CA125 is expressed in ≥80% of nonmucinous epithelial ovarian carcinomas as well as in most endometriod, clear cell and serous carcinomas of the ovary. As stated earlier, CA125 is used in the clinic to follow-up on uterine tumors which show a >60% elevation in levels as well as benign tumors and endometriosis [99].

Chen *et al.* [107] fabricated an electrochemical immunosensor for CA125 that is based on a sensitive mediator, tris (2,2’-bipyridyl) cobalt (III) [Co(bpy)_3_]^3+^ being incorporated into MWNTs-Nafion composite film via ion-exchange. Colloidal gold nanoparticles were then attached onto the composite film through electrostatic interactions. Anti-CA125 antibodies were assembled onto the gold nanoparticle as the biorecognition component in the immunosensor. Electrochemical impedance spectroscopy (EIS) and cyclic voltammetry (CV) were used to characterize the immunosensor fabrication process. The immunosensor had a low detection limit of 0.36 U/mL CA125 and a wide dynamic range consisting of two linear parts ranging 1.0 to 30 U/mL and 30 to 150 U/mL. It also had good reproducibility and stability.

Fu developed a new amperometric immunosensor for the detection of CA125 by immobilization of anti-CA125 Abs on gold hollow microspheres and porous polythionine modified glassy carbon electrodes [108]. The gold hollow microspheres provided a biocompatible microenvironment for proteins, and greatly amplified the number of anti-CA125 molecules immobilized on the electrode surface due to larger surface area. Under optimal conditions, the linear range was from 4.5 to 36.5 U/mL CA125 with a detection limit of 1.3 U/mL.

#### 3.1.2. Cancer Antigen (CA15-3)

CA15-3 (Mucin 1) is carbohydrate antigen that has been used as a biomarker for diagnosis of endometriosis, endometrial carcinoma, and ovarian cancer. This antigen corresponds to polymorphic epithelial mucin sequences (PEMs) which are often overexpressed on the surfaces of malignant glandular cells such as those seen in breast cancer. They are usually shed into the circulation, thus making them useful tumor markers. CA15-3 is present in adenocarcinomas of the breast, colon, lung, ovary and pancreas. It is a more sensitive and specific marker for the monitoring of patients with metastatic breast cancer and levels rise with higher stages of breast cancer. CA15-3 is also used to predict adverse outcomes in this population of patients. However, its usage in detecting breast cancer is limited because of its low sensitivity (23%) and specificity (69%) and because CA15-3 levels can increase in the presence of hepatic cirrhosis, chronic hepatitis, smokers, lupus, tuberculosis and sarcoidosis. Of note, CA27.29 (See Table 2), another mucin marker associated with the gene (Mucin 1) is a slightly more sensitive breast cancer marker than CA15-3 and so the FDA has approved both cancer antigens for monitoring therapy in recurrent or advance breast cancer [96].

Li *et al.* [109] constructed a reagentless and mediatorless immunosensor based on the direct electrochemistry of glucose oxidase (GOx) double layer for determination of CA15-3 antigen. They prepared a composite material containing CNTs and core-shell organosilica@chitosan nanospheres which was then directly cast on the surface of a glassy carbon electrode. Then, Pt nanoclusters (Pt NC) with the role of electron relay were deposited on the surface-modified electrode to form a biocompatible interface with large surface area for the adsorption of the first GOx layer. Then, the second Pt NCs layer was deposited on the surface of GOx to capture anti-CA15-3 Abs. Finally, GO*x* was used as a blocking agent for any remaining open active sites of the Pt NCs to minimize the nonspecific adsorption of other species. The immobilized GOx enzymes showed direct electron transfer with a rate constant of 4.89 s^−1^. The peak current decreased linearly with increasing logarithm of CA15-3 concentration from 0.1 to 160 U/mL with a relatively low detection limit of 0.04 U/mL.

Li *et al.* [110] described a novel, simple, label-free electrochemical immunosensor with N-doped graphene-modified electrode for label-free detection of CA15-3. The immunosensor incorporated a highly conductive graphene (*i.e.*, N-doped graphene sheets)-modified electrode which exhibited significantly increased electron transfer and high sensitivity toward CA15-3. The immunosensor had a low detection limit of 0.012 U/mL and a broad linear range of 0.1–20 U/mL.

Recently, Taleat *et al.* [111] described an electrochemical aptamer-based biosensor for CA15-3 protein detection by using methylene blue (MB) as electrochemical indicator and modifying the graphite-based screen printed electrodes (SPE) detector surface using electropolymerization of a functionalized conductive polymer o-aminobenzoic acid (o-ABA). The primary, capture antibodies of anti-CA15-3 were directly immobilized on the poly o-ABA modified electrodes. Then, a sandwich-like structure was formed upon CA15-3 protein–aptamer complex formation, exploiting the aptamer as the biological detection probe and methylene blue as the electrochemically active marker interacting with the aptamer. Aptamers were used instead of antibodies as the second biological recognition component in this novel immunosensor. The immunoreactions and aptamer binding event were verified via monitoring the interfacial electron transfer resistance with electrochemical impedance spectroscopy (EIS) and by cyclic voltammetry (CV). Finally, CV and differential pulse voltammetry (DPV) were used to detect the change of MB oxidization peak current which was proportional to the human CA15-3 concentration. The biosensor with DPV detection was reliable and sensitive for quantification of CA15-3 with a dynamic range of 1–12 ppb and a detection limit of 0.62 ppb [111].

#### 3.1.3. Carcinoembryonic Antigen (CEA)

CEA, an acidic glycoprotein with a molecular weight of 200 kDa, is the first of the carcinoembryonic proteins and was discovered in 1965 by Gold and Freedman. It remains the most commonly used tumor marker for gastrointestinal cancer. Originally thought to be a specific marker for colorectal cancer, CEA was found to be a nonspecific marker which could be elevated in breast, ovarian, lung and liver cancers for example. It is used to monitor cancer recurrence after surgery and to follow patients during therapy. The serum CEA levels are typically below 5 ng/mL in healthy individuals. CEA studies have revealed an association between highly elevated marker levels, metastases and poor prognosis. Of note, elevated CEA levels prior to colon cancer resection may suggest a worse prognosis. In addition, declining levels during therapy suggest response to therapy, while increasing levels suggest disease progression. However, clinical decisions regarding disease management is not based on CEA levels alone. Since CEA is metabolized in the liver, damage therein can lead to elevated levels in the circulation and therefore false positive results. In addition, CEA levels can be elevated in some patients after radiation and chemotherapy. Despite these limitations, CEA is used as a marker for monitoring colorectal cancer, though its use as a screening tool in asymptomatic patients is limited by a low positive predictive value for diagnosis of gastrointestinal cancer [99].

Traditionally, CEA levels have been monitored in clinical settings by using methods such as fluoroimmunoassay [112], enzyme immunoassay [113], and radioimmunoassay (RIA) [114]. Although sensitive and selective, these immunoassays have long analysis times, require skilled personnel and are difficult to automate. RIAs also expose laboratory workers to radioactive labels that are a potential safety hazard.

Gao *et al.* [115] constructed an amperometric immunosensor for detection of CEA utilizing a uniform CNT-based film fabricated via layer-by-layer (LBL) assembly of positively charged CNTs wrapped by poly(diallyldimethylammonium chloride) and poly(sodium-*p*-styrene-sulfonate, a negatively charged polymer). This nanomultilayer film resulted in a high surface area that is accessible to sample components and a biocompatible microenvironment for the portion of the immunosensor that is in contact with the sample. This was followed by electrodeposition of gold nanoclusters on the modified glassy carbon electrode to which anti-CEA antibodies were finally immobilized. The steps in the fabrication process and the electrochemical performance of the immunosensor were characterized using CV, electrochemical impedance spectroscopy (EIS), and scanning electron microscopy (SEM). This immunosensor, utilizing amperometric detection, had a detection limit of 0.06 ng/mL and linear ranges from 0.1 to 2 ng/mL and from 2 to 160 ng/mL [115]. The sensor also had good long-term stability with 95.6% and 82.5% current responses after 20 and 30 days of storage respectively. In addition, the immunosensor could also be regenerated in about 5 min by soaking the probe in either urea or glycine-hydrochloric acid. The ability to regenerate an immunosensor is a crucial factor in any biomarker detection application intended for repeated measurements in a clinical laboratory setting.

Yang *et al.* [88] developed an electrochemical immunosensor for detecting CEA using three-dimensional gold-titanium oxide (Au-TiO_2_) nanoparticles assembled on a glassy carbon electrode to which the anti-CEA capture antibodies were immobilized as the sensor platform with the assistance of functionalized hollow Pt nanophere bioconjugates as labels. The hollow Pt nanospheres containing the secondary antibody capable of binding CEA and the horseradish peroxidase enzyme labels were incubated with the sensor after the immunoreactions between anti-CEA capture Ab and the CEA biomarker had taken place. The immunosensor was tested in human serum samples and compared with an ELISA method with relative standard deviations of less than 5% between the two methods at three different concentrations of CEA. The immunosensor had detection limit of 12 pg/mL and a wide linear range from 0.02 to 120 ng/mL CEA [88]. The immunosensor was also very stable with 91.1% of initial response after storing it in PBS buffer for 21 days.

#### 3.1.4. Prostate Specific Antigen (PSA)

PSA is a serine protease that is synthesized specifically in the epithelial cells of the prostate gland and its expression therein is regulated by the androgen receptor. Its high tissue specificity makes it one of (if not the) most widely used tumor marker. The normal reference range for PSA is 0–4 ng/mL and its cancer sensitivity as well as its tissue specificity makes PSA the most useful tumor marker available for screening and managing prostate cancer. However, its main drawback is the lack of specificity in distinguishing prostate cancer from nonmalignant prostate disease. Indeed, benign conditions such as benign prostatic hypertrophy, acute prostatitis, and infarction may be correlated with elevated PSA levels [99]. PSA can be bound to protein or free, the sum of which gives the total PSA which is the most commonly ordered test. As stated earlier, (total) PSA can be elevated in prostate cancer as well as prostate enlargement, prostate inflammation, in microscopic and/or clinically insignificant cancers and with leaked of prostatic fluid into the circulation. These potential interferences necessitate the use of age-corrected PSA norms. For example, free PSA is only performed when the total PSA gives an equivocal result (between 4 and 10 ng/mL). Of note, there are strategies to increase the sensitivity and specificity of PSA testing. These include; the use of PSA velocity (rate of PSA increase over time) wherein an increase of 2 μg/L in a 1-year period predicts the presence of an aggressive cancer, the use of PSA doubling time (time for a PSA value to double) and PSA density (PSA concentration divided by the prostate volume as measured by ultrasound). Complexed PSA (cPSA) and free PSA (fPSA0 are also used to distinguish prostate cancer from other causes of PSA elevation. Of note, men with prostate cancer have elevated levels of cPSA and low levels of fPSA. However, the gold standard for prostate cancer diagnosis is tissue biopsy. Although dependent on age, total PSA levels >8 ng/mL may indicate the presence of prostate cancer [100].

Wan *et al.* [38] fabricated a CNT-based, multiplexing, electrochemical immunosensor utilizing sandwich-immunoassay type strategy on disposable screen-printed carbon electrode (SPCE) array detection platform for sensitive and simultaneous determination of PSA and interleukin 8 (IL-8), another cancer biomarker. The capture antibodies (Abs) were covalently immobilized on SPCE array by first electrochemically activating the carbon working electrode to generate carboxylate groups. These groups were then used for covalent attachment of capture Abs via their amine residues. The secondary Abs and the Horseradish peroxidase (HRP) labels were immobilized on MWNTs by using similar attachment chemistry. The detection limit of PSA was 5 pg/mL and 8 pg/mL for IL-8 [38].

Wang *et al.* [116] recently prepared a sensitive and selective, label-free electrochemical immunosensor for PSA based on silver hybridized mesoporous silica nanoparticles. Mesoporous silica nanoparticles (MSNs) were used to increase the surface area and hence the capacity to immobilize the primary antibody and silver nanoparticles (Ag NPs) to enhance the electron transfer rates. The silver hybridized mesoporous silica nanoparticles were synthesized and used as the electrode material. Hydroquinone was used as a mediator and helped generate a stable electrochemical signal. Based on the specific and selective antibody–antigen binding interaction, a label-free immunosensor was developed with a wide dynamic range from 0.05 to 50.0 ng/mL and a detection limit of 15 pg/mL [116]. The immunosensor was also used to determine PSA in human serum samples with satisfactory results.

3.1.5. α-Fetoprotein (AFP)

AFP is a fetal serum protein and also one of the major carcinoembryonic proteins. It resembles albumin in physical and chemical aspects. In the fetus, AFP is synthesized by the yolk sac, the fetal hepatocytes and to a lesser extent by the fetal gastrointestinal tract and kidneys. Elevated AFP is found in patients with primary hepatocellular carcinoma (HCC) and yolk-sac derived germ cell tumors. Therefore, it is the most useful serum marker for the diagnosis and management of HCC and germ cell tumors. However, AFP is transiently elevated in during pregnancy and in many benign liver diseases. Due to the high prevalence of liver cancer in China and other countries in south-east Asia, AFP testing is used in screening for HCC in that region of the world. In addition, tests for both AFP and human chorionic gonadotropin (hCG) have been helpful in reducing clinical staging errors in patients with some testicular tumors and aid in the differential diagnosis of various germ cell tumors. In terms of detecting HCC in patients with hepatitis B and C, studies indicate that combined screening with AFP and ultrasonography results in increased sensitivity from 75% to approximately 100%. Of note, AFP is offered during prenatal screening for neural tube defects (and in conjunction with free β-hCG and unconjugated estriol) for Down syndrome [99].

Du *et al.* developed a novel, selective immunosensor incorporating a graphene sheet sensor platform and colloidal carbon nanospheres (CNSs) synthesized from fructose labeled with HRP-secondary antibodies (HRP-Ab_2_) via EDC/NHS (ethyl(dimethylaminopropyl) carbodiimide/*N*-Hydroxysuccinimide) immobilization for the sensitive detection of AFP [117]. The immunosensor had enhanced sensitivity for AFP as a result of a dual signal amplification strategy where: (1) multi-enzyme labeling based on the synthesized homogeneous CNSs with a narrow size distribution which were each able to bind several HRP-Ab_2_ complexes forming multi-bioconjugates of HRP-Ab_2_-CNSs that were introduced onto the electrode surface; and (2) using functionalized graphene sheets with very large surface areas capable of capturing a very large amount of primary (aka capture) antibodies (Ab_1_) to modify a pretreated carbon electrode surface. The incorporation of a nanomaterial with fast electron transfer kinetics (*i.e.*, graphene modification of a carbon electrode) and CNS multi-enzyme labeling provided a seven-fold increase in the signal detected by cyclic voltammetry (CV) and square wave voltammetry (SWV). This immunosensor had detection limit of 0.02 ng/mL and a linear range from 0.05 to 6 ng/mL.

Su *et al.* [118] prepared a new sandwich-type electrochemical immunsensor for detection of AFP in serum using nanogold-enclosed titania nanoparticle (AuTi)-labeled secondary Ab on a gold-silver-graphene hybrid nanosheet-functionalized glassy carbon electrode. The inclusion of the hybrid nanosheets increased the immobilized amount of biomolecules and improved the electrochemical performance of the immunosensor. The use of AuTi nanolabels significantly amplified the electrochemical signal and the electrochemical properties of the immunosensing interface more when compared to using pure nanogold or titania-based labels. The immunosensor, utilizing amperometric detection, had a low AFP detection limit of 0.5 pg/mL and a wide dynamic range from 0.001 to 200 ng/mL [118]. Satisfactory immunsensor stability was obtained with about 90% of the original peak current still achievable after storing the device for 13 days.

Tang *et al.* [119] described the fabrication and testing of a simple and sensitive sandwich-type conductometric immunosensor for detection of AFP in serum specimens utilizing carbon nanoparticles as labels [119]. The immunosensor utilizing conductometric detection had an AFP detection limit of 50 pg/mL and a wide dynamic range from 0.1 to 500 ng/mL.

### 3.2. Other Non-Cancer Antigen Biomarkers with Relevance in Other Medical Fields (Specifically Psychiatry and Behavioral Science)

#### 3.2.1. Cortisol

Cortisol is a steroid hormone released by the adrenal glands in response to adrenocorticotropic hormone (ACTH) stimulation from the anterior pituitary gland. It is a monovalent antigen due to its low molecular weight of 362.47 g/mol. Cortisol has significant effects, especially on carbohydrate metabolism. Its level in the circulation normally peaks at in the morning hours, drops in the evening and reaches a nadir around midnight. Cortisol can be obtained from the blood, saliva and urine to diagnose Cushing Syndrome (hypercortisolism), Addison disease (hypocortisolism), monitoring treatment for hypercortisolism or hypocortisolims and evaluating stress-related conditions. When testing for conditions involving excessive release of cortisol, the peak serum level may be normal, but the diurnal drop may not be observed. As a result, levels should be drawn at 8 AM, 4 PM and sometimes 8 PM. When testing for conditions involving decreased cortisol release, a single morning level may be drawn. To distinguish Cushing syndrome from simple obesity, a 24 h urine free cortisol level may be appropriate. Urine free cortisol is proportional to serum free cortisol and is also the most sensitive and specific test for the initial screening for Cushing syndrome. Cortisol is also present in saliva and is proportional to unbound cortisol in the serum, including diurnal variations. Thus, in adrenal insufficiency (as can be found in Addison disease among others), the morning salivary cortisol is decreased, while evening salivary cortisol levels (drawn between 11 PM and midnight) are increased in Cushing syndrome. Of note, cortisol reference ranges may vary by time drawn (whether in adults, adolescents or children), by site (serum, saliva or urine), by methodologies (radioimmunoassay or high-performance liquid chromatography). Extremely high cortisol levels in the morning in addition to a lack of diurnal variation may suggest the presence of carcinoma. Also, certain drugs are associated with increased cortisol levels, such as cocaine, amphetamines, alcohol, nicotine and naloxone, while lithium, levodopa, phenytoin (in women), morphine, etomidate and ketoconazole can cause decreased cortisol levels. Cortisol levels may also be increased in bright light exposure, ectopic ACTH production, high stress (thermal, traumatic or physiological), metabolic syndrome (with hypertension and obesity), burns, shock, post-surgical states, severe liver or kidney disease, acute infections or inflammatory disease, hyperpituitarism, hyper thyroidism, pregnancy, strenuous exercise, hypoglycemia, and drugs (coticotropin, estrogens, oral contraceptives, yohimbine and vasopressin). Decreased cortisol levels may also be seen in chromophobe adenoma, craniopharyngioma, hypothyroidism, liver disease, postpartum pituitary necrosis, rheumatoid arthritis and drugs (dexamethasone). Organophosphate exposure on the central nervous system can cause alterations in the endocrine system that can lead to imbalances in cortisol secretion which may then be measured by immunosensors and immunoassays. In psychiatry and behavioral science, cortisol has a regulatory effect on serotonin function and a stimulant-like effect on dopamine neurotransmission. In general, excessive cortisol activity may contribute to symptoms of psychotic mood disorders and schizophrenia, while cortisol levels may be elevated in brief or sustained stress and major depression. In contrast, studies have shown that cortisol levels are low in post-traumatic stress syndrome (PTSD). Furthermore, stress and cortisol elevation have been implicated in studies of relapse in substance-dependent patients [100].

Unlike with some other biomarkers, with cortisol (which is linked with many stress-induced diseases and requires near real-time detection), the model where the biological fluid samples are first collected from patients with later processing and analysis in centralized laboratories with relatively long reporting times and a possibility of several points where quality control could fail, does not work well. Therefore, much effort in the recent years has gone towards the development of easy-to-use biosensors for on-site measurement of cortisol levels and related diagnosis.

Nanomaterials, such as CNTs and conducting polymer-metal nanocomposites have been used in cortisol sensing as the chemiresistive transducer and as the antibody immobilization matrix respectively, to enhance the sensitivity and selectivity of electrochemical cortisol immunosensors. Tilli *et al.* [120] fabricated a label-free immunosensor for cortisol using SWCNTs as the chemiresistive transducer. The SWCNTs were functionalized with cortisol-3-O-carboxymethyloxime-N-hydroxysuccinimide (3-O-CMO-NHS) ester, a cortisol analog, and a monoclonal anti-cortisol antibody was ligated to this receptor. Cortisol from artificial saliva displaced the anti-cortisol antibody at the receptor producing corresponding decreases in the resistance/conductance of the nanotube-biomolecule hybrid biosensor. The immunosensor was selective and sensitive with detection limit of 1 pg/mL, a sensitivity of 14.9 ng/mL and a linear range from 1 pg/mL to 10 ng/mL.

Arya *et al.* [121] prepared a mediator and label free cortisol biosensor by depositing polyaniline (PANI) protected gold nanoparticle composite electrophoretically onto a gold electrode. Monoclonal antibodies specific and selective for cortisol were then covalently immobilized onto the surface of the conducting polymer/Au electrode using N-ethyl-Nʹ-(3-dimethylaminopropyl) carbodiimide and *N*-hydroxysuccinimide (EDC/NHS) chemistry. Using cyclic voltammetry as the electrochemical detection method, they obtained a detection limit of 1 pM, a sensitivity of 1.63 μA/M and a linear range from 0.36 pg/mL to 36 ng/mL (1 pM–100 nM). They also explored a similar biosensor design with the PANI polymer nanocomposite containing silver/silver oxide (Ag/AgO) (core/shell with diameter = 5 nm). This immunosensor had a detection limit of 0.64 pM/mL, a sensitivity of 0.66 μA/M and a linear range from 0.36 pg/mL to 3.6 μg/mL.

Yamaguchi *et al.* [122] designed a reusable immunosensor for noninvasive and quantitative analysis of cortisol in saliva in about 35 minutes. The immunosensor has a fluid control mechanism with both a vertical and a lateral flow. The immune reaction, a competitive immunoassay, occurs in the vertical flow component of the sensor while the lateral flow component removes proteins, other steroid hormones, and any unreacted enzyme-labeled cortisol-conjugate from the sample thereby minimizing cross-reactivity of the anti-cortisol Abs and ultimately improving the sensitivity of the biosensor. A flat electrode consisting of three Pt electrodes (a working electrode, a reference, and an auxiliary electrode) detects the current produced by the oxidation of hydrogen peroxide which is previously produced by the glucose oxidase enzyme label in the presence of glucose substrate. This immunosensor with fluid control mechanism had a dynamic range from 0.1–10 ng/mL cortisol in saliva [122]. Typical cortisol concentration range in the saliva of healthy adults is 1–8 ng/mL [123].

#### 3.2.2. Ceruloplasmin (Cp)

Serum ceruloplasmin (along with serum copper and calculated free serum copper) is used to screen for the autosomal recessive inherited disorder of copper metabolism in which circulating free copper levels are elevated leading to excess copper being deposited in the brain, eyes, liver and kidneys known as Wilson disease (Hepatolenticular degeneration). In Wilson disease, serum Cp is decreased, while the free copper level is elevated (>25 μg/dL) [100]. Cp is a copper-binding protein which is synthesized in the liver with a molecular weight of 132 kiloDaltons (kDa) and consists of a single polypeptide chain. Serum levels are 20–40 mg/dL in normal adults which may be elevated twofold in oral contraceptive therapy, pregnancy or as an acute phase reactant (a marker of inflammation, infection, tissue injury, malignancy and cardiovascular disease) [99,100]. Drugs associated with elevated Cp levels include anticonvulsants, estrogens, methadone and nicotine, while drugs associated with elevated copper levels include anticonvulsants and estrogens. A molecule of Cp can bind six atoms of copper imparting a blue color to the protein and combined with the yellow of other chromogens of plasma imparts a greenish color to plasma with elevated Cp [99]. Screening for Wilson disease is indicated if there is clinical suspicion of Wilson disease, family history of Wilson disease, early onset hepatitis or cirrhosis and neuropsychiatric symptoms consistent with Wilson disease. Diagnosi of Wilson disease is confirmed based on physical findings (liver disease, neurologic signs, Kayser-Fleischer ring in the cornea), low serum Cp level, increased serum free copper, increased serum copper and increased urine copper [99,100]. Neuropsychiatric symptoms of Wilson disease may precede other disease manifestations and present as psychosis (delusions and hallucinations), depression, mild cognitive impairment, behavioral problems (personality changes), and abnormal motor system movements (parkinsonism, dystonia, chorea, tremor (the classic “wing-beating” type), lack of coordination, hypokinetic speech, dysphagia, bulbar and pseudobulbar palsies [100].

Ojeda *et al.* [124] prepared and described the first electrochemical immunosensors for determination of Cp in 2013. Two configurations involving magnetic beads (MBs) functionalized with Protein A or Streptavidin for immobilization of Cp Abs were compared using competitive immunoassay format with synthesized alkaline phosphatase (ALP)-Cp conjugate. ALP is the enzyme label responsible for catalyzing the formation of electrochemically active 1-naphthol from 1-naphthylphosphate substrate. Upon capturing MBs-immunoconjugates onto screen-printed electrodes (SPEs), quantification of Cp was completed by differential pulse voltammetry (DPV) detection of 1-naphthol produced in response to 1-naphthylphosphate addition. Linear range of calibration curve for Protein A-MBs was 0.1–1000 µg/mL with a detection limit of 0.040 µg/mL. The corresponding linear range for Streptavidin immobilized anti-Cp Abs on MBs was 0.025–20 µg/mL with a 0.018 µg/mL detection limit [124]. Good results were also obtained when using the immunosensor for the determination of Cp in spiked human serum samples.

Recently, Garcinuño *et al.* [125] of the same research group, described a novel amperometric immunosensor for the determination of Cp in human serum and urine based that is based on covalent binding to CNT-modified screen-printed electrodes. This is the first reported immunosensor for Cp with amperometric detection. The immunosensor configuration includes an indirect competitive immunoassay with covalent immobilization of Cp, the analyte, on activated carboxylic acid groups at CNT-modified screen-printed electrodes (SPE). After Cp immobilization, an immunoreaction is allowed to take place between the free Cp from the sample and anti-Cp Abs in solution and the remaining non-conjugated anti-Cp Ab is attached onto the Cp-CNTs modified SPE. Ultimately, the determination of Cp is done by adding a secondary antibody labeled with horseradish peroxidase (HRP) anti-IgG and measuring the amperometric current resulting from the addition of hydrogen peroxide (H_2_O_2_) in the presence of hydroquinone as the redox mediator. The immunosensor for Cp had good reproducibility, a linear range between 0.07 and 250 μg/mL and the detection limit was 21 ng/mL [125]. The analytical performance of the immunosensor should allow the fast determination of Cp in spiked human serum and urine samples.

#### 3.2.3. Thyroid-Stimulating Hormone (TSH)

TSH is a glycoprotein hormone produced by thyrotrope cells in the anterior pituitary gland. TSH stimulates the thyroid gland to release thyroxine (T_4_) and stored triiodothyroxine (T_3_) which are hormones that determine basal metabolic rate, protein synthesis, carbohydrate, lipid and vitamin metabolism and calcium release from bones. In addition, T_4_ is converted to the more potent T_3_. The circulating T_4_ and T_3_ influence the release of TSH and thyrotropin-releasing hormone (TRH) from the hypothalamus via a feedback inhibition. Thyroid function can be tested by: (1) measuring free thyroxine (free T_4_, FT_4_); or (2) by measuring TSH (sensitive TSH assay/highly sensitive TSH assay or thyrotropin assay). FT_4_ is measured in preference to total T_4_ because its measurement is not affected by blood protein levels. Blood is usually required for the test and FT_4_ is usually ordered after an abnormal TSH test or when clinical suspicion of thyroid disease persists even though TSH is within normal levels. In fact, variations in levels of TSH and FT_4_ together suggest the following disease states:

TSH ↑, FT_4_ ↓: hypothyroidism;
TSH ↑, FT_4_ normal: subclinical hypothyroidism;
TSH ↓, FT_4_ ↑: hyperthyroidism;
TSH ↓, FT_4_ normal: subclinical hyperthyroidism;
TSH ↓, FT_4_ ↓: nonthyroidal illness.

FT_4_ is measured when there is an abnormal TSH result or signs/symptoms of thyroid disease (in the presence of normal TSH). Reference range for TSH in adult human serum is usually between 18 pg/mL and 0.23 ng/mL. The exact linear detection range depends on the assay methodology, therefore it is recommended to consult laboratory reference values. However, if patients are already being treated for hypothyroidism using levothyroxine, the upper limit of normal is 0.05 ng/mL. Increased levels of FT_4_ suggest hyperthyroidism or treated hypothyroidism, while decreased FT_4_ levels suggest primary hyperthyroidism (due to thyroid gland dysfunction), secondary hypothyroidism (due to a pituitary cause), tertiary hypothyroidism (due to a hypothalamic cause), hypothyroidism treated with triiodothyronine (T_3_) and late pregnancy. Hyperthyroidism, as defined by the low TSH and elevated FT_4_ levels, may present with psychiatric symptoms such as mood disorders, psychosis and delirium. In the elderly, a syndrome of apathetic hyperthyroidism may be present and is characterized by lethargy, tachycardia, atrial fibrillation, heart failure and severe cognitive impairment which may be diagnosed as dementia [100]. On the other hand, hypothyroidism, as defined by low FT_4_ with elevated TSH levels, is associated with fatigue, depression, and memory impairment, while low FT_4_ with low TSH is seen in nonthyroidal illness in acutely ill patients. A normal FT_4_ with an elevated or low TSH level suggests subclinical hypothyroidism or hyperthyroidism respectively which may manifest into fully developed or attenuated thyroid disease. As noted earlier, FT_4_ can vary in pregnancy, severe or chronic illness. In fact, dated FT_4_ may also be caused by certain drugs such as aspirin, furosemide, heparin, carbamazepine, phenytoin, propranolol, valproate and contrast material used in medical imaging, while drugs that cause low FT_4_ levels include phenytoin, methadone, phenobarbital, estrogen, lithium, carbamazepine and oral contraceptives [100].

Measuring thyroid function via TSH (sensitive TSH assay/highly sensitive TSH assay or thyrotropin assay) is useful when pituitary (secondary) hypothyroidism is suspected. In this case, TSH should be tested along with FT_4_. If the thyroid itself (primary disease) is suspected to be the source of hyper or hypothyroidism, then the sensitive TSH is the best initial screening test. If the assay is normal, no further testing is required. However, if the assay is not normal, FT_4_ is measured. TSH and FT_4_ together suggest the following diagnoses:

TSH ↑, FT_4_ ↓: hypothyroidism;
TSH ↑, FT_4_ normal: subclinical hypothyroidism (and check thyroid antibodies);
TSH ↓, FT_4_ ↑: hyperthyroidism (thyroid-stimulating immunoglobin, thyroid peroxidase;
(TPO) antibody, and TSH receptor antibody are checked);
TSH ↓, FT_4_ normal: subclinical hyperthyroidism;
TSH ↓, FT_4_ ↓: nonthyroidal illness.

Indications for testing, include: diagnosing hypo- or hyperthyroidism in a symptomatic patient, screening for thyroid disease in at-risk patients (such as women over 50 years old), patients with newly diagnosed type 2 diabetes, pregnant women, postpartum women and patients treated with lithium, monitoring the efficacy of thyroid replacement therapy in a patient with hypothyroidism, standard component of the workup for patients with depression, memory impairment, or dementia and assisting in the work up of female infertility. Reference ranges vary by age, type of hyper- or hypothyroidism (borderline and probable). Treatment for hyper- or hypothyroid states may cause TSH to remain abnormal for up to 6 weeks after a euthyroid state has been reached. Critical values of <0.1 μU/mL indicates primary hyperthyroidism or exogenous thyrotoxicosis and risk of atrial fibrillation (a major risk factor for stroke). Increased levels of TSH can be caused by primary hypothyroidism (up to 100 times normal), TSH-producing tumor (e.g., breast or lung), Hashimoto thyroiditis, the recovery phase of subacute thyroiditis or nonthyroidal illness, insufficient thyroid replacement or thyroid hormone resistance treated hypothyroid patients. Decreased levels of TSH may be due to primary hyperthyroidism, secondary hypothyroidism (pituitary disease), tertiary hypothyroidism (hypothalamic disease), subclinical hyperthyroidism (due to toxic mutlinodular goiter or treated Graves disease), euthyroid sick syndrome or over-replacement of thyroid hormone in treated hypothyroid patients. Other factors that can affect TSH levels include old age (>80 years old, upper limit of normal is 10 μU/mL), treatment with atenolol, carbamazepine, aripiprazole, estrogen, chlorpromazine, ferrous sulfate, haloperidol, lithium, metoclopramide, iodine-containing food or drugs, morphine, lovastatin, phenothiazines, prednisone, phenytoin, sumatriptan, valproate, and amphetamine (abusers). Decreased TSH levels can be caused by acute illness, extreme stress, aspirin use, corticosteroids, carbamazepine, interferon-α2, hydrocortisone and the first trimester of pregnancy. Of relevance to psychiatry and behavioral health, low TSH with elevated FT_4_ indicates hyperthyroidism which can be associated with mood disorders, psychosis, and delirium. In the elderly, a syndrome of apathetic hyperthyroidism may be present and is characterized by lethargy, tachycardia, atrial fibrillation, heart failure and severe cognitive impairment which may be diagnosed as dementia [100]. Elevated TSH with low FT_4_ indicates hypothyroidism which is associated with fatigue, depression, and memory impairment. Low TSH with low FT_4_ is commonly seen in acutely ill patients, while abnormal TSH with normal FT_4_ is consistent with subclinical thyroid disease which may develop into full-blown hyper- or hypothyroidism or an attenuated form of these two entities. Of note, numerous psychotropic drugs affect TSH level (as detailed above) without causing disease [100].

Zhang *et al.* [126] designed a novel electrochemical immunosensor for sensitive determination of TSH utilizing nanogold-functionalized magnetic beads (GoldMag) on a gold nanoparticle-dispersed graphene nanosheets immunosensing platform. The authors prepared polyethyleneimine-functionalized magnetic beads by a wet chemical method onto which electroactive thionine molecules and gold nanoparticles were alternately immobilized using and opposite-charged adsorption technique and an *in situ* synthesis method, respectively. The GoldMag nanostructures served as signal trace tags for the enzyme label horseradish peroxidase (HRP)-anti TSH antibody conjugates in a sandwich immunoassay. The conjugated signal tags increased with increasing TSH concentration which was also determined in human serum specimens. This electrochemical immunosensor had detection limit of 0.23 pg/mL and a linear range from 0.45 pg/mL to 0.91 ng/mL [126]. The results indicated that the GoldMag nanostructures, conjugated with HRP, enhanced the sensitivity of the electrochemical immunosensor. The signal amplification was partially achieved by high HRP-loading on the nanostructures and in part the high conductivity of the nanomaterials.

#### 3.2.4. Luteinizing Hormone (LH)

In response to stimulation by gonadotropin-releasing hormone (GHR) from the hypothalamuc, LH is secreted by the anterior pituitary which in turn stimulates the testes to produce testosterone and the ovaries to produce estradiol. These hormones then feed back to the hypothalamus and pituitary to help regulate LH release. In females, a surge of LH in mid-menstrual cycle causes ovulation of the follicle-stimulating hormone (FSH)-ripened ovarian follicle. Therefore, in women, LH and FSH levels can help distinguish failure of the ovaries themselves (primary ovarian failure) from ovarian failure due to pituitary or hypothalamic dysfunction (secondary ovarian failure). LH and FSH may also be utilized in the diagnoses of virilization/hirsutism, polycystic ovary syndrome, reduced sex drive, infertility, and precocious or delayed puberty. In primary ovarian failure, both LH and FSH levels are high. In males, LH and FSH are needed for sperm development and maturation. In addition in males, LH and FSH may be indicated for testing in hypogonadism, testicular tumors, cryptorchidism, reduced sex drive, infertility and erectile dysfunction. It is important to note that LH and FSH levels fluctuate throughout the day; therefore a single sample may give inaccurate hormone activity.

Reference ranges differ by laboratories and by stage of puberty, gender, and menstrual cycle phase. As stated earlier, increased levels are seen in primary ovarian failure, primary testicular failure, polycystic ovary syndrome, menopause, precocious puberty and liver disease, while decreased levels are seen in pituitary or hypothalamic dysfunction resulting in secondary gonadal (ovarian or testicular) failure, anorexia nervosa, depression, severe stress, malnutrition, severe illness and delayed puberty. LH levels can also be increased with the use of naloxone, spironolactone, clomiphene or certain anticonvulsants, while decreases may be noted with the use of phenothiazines, oral contraceptives, digoxin, or hormone treatments. Of note, LH levels are used in the work up of certain psychiatric and behavioral conditions. For example, LH levels (as stated earlier), may be decreased in anorexia nervosa, severe stress, phenothiazine use or depression. LH levels may be increased with naloxone use [100].

Lillie *et al.* [127] fabricated a simple immunosensor for LH measurement between 1 and 800 IU/L using impedance spectroscopy detection. The sensor was prepared by polymerizing pyrrole loaded with antibody for LH on a gold inter-digitated electrode. Farace *et al.* [128] also prepared an immunosensor for LH with impedance spectroscopy detection. The reagentless sensor had intergrated biorecognition and transduction system with the antibodies immobilized by entrapping them in a conducting polypyrrole matrix.

## 4. Nanomaterials and the Use of Nanotechnology for Clinical Diagnostic Purposes

The use of nanotechnology and the variety of unique nanomaterials with favorable electrochemical and surface properties that were described above, has helped: (1) maximize the detection capabilities by improving the signal-to-noise ratios; (2) improve selectivity and minimize interference from biological specimens; and (3) increase the stability of the biosensors and related reagents to a standard where they meet the demands for detection of biomarkers at extremely low concentrations (typically pg/mL to ng/mL). Known nanomaterials and their application in the detection of cancer and disease management in other fields of medicine are summarized in Table 3 below.

**Table 3:** Nanomaterials and their applications in medicine.

Nanomaterials	Potential Applications in Cancer Detection	Ref.
Au-Ag-graphene hybrid nanosheets	Detection of alpha fetoprotein (AFP)	[118]
Au-nanowires dopes Sol-Gel film	Detection of testosterone	[129]
Au-TiO_2_ nanoparticles with Pt nanophere bioconjugates	Detection of carcinoembryotic antigen (CEA) in breast cancer	[88]
[Co(bpy)_3_]^3+^ in MWNTs-Nafion composite film and Au NPs	Detection of ovarian and uterine cancer by CA125 biomarker	[107]
Chitosan-CNTs-AuNPs nanocomposite film	Detection of carcinoembryotic antigen (CEA)	[130]
CNTs and core-shell organosilica@chitosan nanospheres	Detection of ovarian cancer by CA125 biomarker	[109]
Graphene	Detection of breast cancer by CA 15-3 biomarker	[110]
Graphene sensor platform with colloidal carbon nanospheres	Detection of alpha fetoprotein (AFP)	[117]
QD-based microfluidic protein chip	Multiplexed detection of CEA and AFP	[47]
NanoAu-functionalized magnetic beads on Au NP-dispersed graphene	Detection of thyroid stimulating hormone (TSH)	[126]
SWCNT conducting polymer-metal nanocomposites	Detection of cortisol	[120]
SWNT forests	Detection of oral cancer biomarker Interleukin-6 (IL-6)	[131]
Silica nanoparticles with silver nanoparticles	Detection of prostate specific antigen (PSA)	[116]

## 5. Electrochemical Detection

One of the greatest challenges in transferring well studied chemical reactions from the macroscopic scale to the micro- and nanoscale is the detection step. Many immunoassays used in clinical analysis rely on spectrophotometric detection that requires rather bulky light sources, monochromators, sample cells with specific path lengths to obtain high sensitivity, and detectors. These methods may also require a fair amount of the sample and may give false positive or negative results due to colored, turbid, and complex sample matrices. Electrochemical detection methods, which are based on interfacial phenomenon, are better suited for detection in small sample volumes (from microliters to as low as nanoliters) as the sensitivity of these methods is independent of the sample volume used in the measurement [2]. Extremely low detection limits may be achieved (zeptomols, 10^−21^ mol) with electrochemical detection [132,133]. In these biosensors, the biological reaction is transformed into a measurable signal by the electroanalytical detection method.

Also, the interferences from other sample components are easier to eliminate in electrochemical methods, for example by carefully choosing the detection potential in amperometry, and the detection can be done in complex colored or turbid samples and can be used in homogeneous immunoassays that are typical in clinical analysis [2]. Additionally, most electroanalytical detection methods require little or no sample preparation prior to analysis. Also, the electrochemical instruments that detect and record the signal tend to be inexpensive and are often portable, even handheld devices.

### 5.1. Electrodes

Most electrodes used in immunosensors are made of different forms of carbon such as glassy carbon, graphene, carbon fiber, or epoxy graphite or alternatively inert metals such as gold or platinum [36]. The choice of electrode not only affects the cost of the assay or biosensor, but also the sensitivity of the detection method. Therefore, the electrode material and design must be optimized for each application. In the cases where the biorecognition molecule such as an antibody is immobilized onto the surface of the electrode, the electrode material also limits the immobilization procedures that can be used [25]. For example, microelectrodes (and more recently nanoelectrodes) have been used in quantification of analytes of biological significance both *in vivo* and *in vitro* [134,135,136,137,138,139,140,141]. In addition, their extremely small sizes make them less invasive in intracellular and extracellular environments. Nanoelectrodes can be advantageous in a multitude of biological applications such as single cells studies, coordinated biosensor development, fabrication of microchips, point-of-care clinical analysis and in patterned electrodes [140,142]. Furthermore, applications using individual nanoelectrodes and nanoelectrode arrays are a rapidly developing research area due to frequent advancements in materials science and recent, more cost effective, electrode fabrication methods. Also, the integration of the electronic and the biological components in the biosensors is critical to the development of highly sensitive miniaturized devices used in medicine for screening and/or diagnosis. Other biosensors include disposable screen-printed electrodes (SPEs) made of graphite, gold, and silver and [38,66,111,124,125] they can easily be mass produced and have low fabrication cost. Also included are interdigitated electrodes (IDEs) which have been used as transducers in biosensors for the determination of TSH [143] as well as interdigitated microelectrodes (IMEs) or interdigitated microelectrode array (IDAs) which are microelectrodes that consists of a pair of opposing metal electrodes of variable digit width and separation ranging from 100 nm to 10 μm. These electrodes are usually microlithographically fabricated on silicon or glass substrates.

### 5.2. Electrochemical Sensors

Electrochemical detection cell typically consists of either two or three electrodes. A two electrode system has only the working and reference electrodes, whereas a three electrode system has the working, reference, and auxiliary (most commonly a platinum wire) electrodes. The working electrode (a.k.a. indicator electrode) is usually made of a solid, conductive material, such as platinum, gold, or glassy carbon. In the three electrode system, the charge from electrolysis passes through the auxiliary electrode (aka the counter electrode) instead of the reference electrode, thereby protecting the reference electrode from changing its half-cell potential against which the electrochemical processes are measured over time. A reference electrode is a known half-cell such as silver/silver chloride (Ag/AgCl) or saturated calomel electrode (SCE) that (1) is insensitive to the sample solution; (2) is reversible; (3) obeys the Nernst equation; (4) provides constant potential throughout the analysis; and (5) finally returns to the original potential.

Generally, electroanalytical techniques measure current, potential or impedance. These techniques fall into four main categories: (1) potentiometry; (2) amperometry; (3) voltammetry; and (4) coulometry. In amperometry, a constant potential (mV) is applied to the sample, while changes in current, *∆i* (A) are measured. Unlike amperometry, in voltammetry, the potential is varied over time, while changes in current *∆i* (A) are monitored. Potentiometry does not involve an oxidation reduction process and measures the cell potential, *E*_membrane_ (V or mV) across a thin selectively permeable membrane. In coulometry, which measures charge (C), the amount of an electroactive analyte can be determined based on a measurement of the total coulombs of electricity needed to quantitatively oxidize or reduce the analyte of interest. 

In this review, amperometry, voltammetry, impedometry and conductometry are described in more detail as these are the four most common electroanalytical methods used in biosensor development, optimization, characterization, and analyte detection. For example, the performance of the newly prepared biosensors incorporating nanomaterials in contact with the working electrode is typically evaluated by cyclic voltammetry. Meanwhile, amperometry and impedometry are commonly used to determine the concentration of biomarkers in biological samples analyzed using electrochemical immunosensors.

#### 5.2.1. Amperometric Sensors

Amperometric detection is one of the most popular electroanalytical detection methods due to its simplicity and the low detection limits that can be achieved by the biosensor. In amperometry, the analyte concentration is determined by measurement of the signal, the current produced in a suitable redox reaction as a function of time when a constant potential is applied to the electrodes. Three-electrode systems with working, auxiliary, and reference electrodes are usually used in amperometry. In amperometry, the oxidation or reduction potential used for the detection step is characteristic of the analyte species adding to selectivity of the method by eliminating interferences from other redox active species that may also be in the sample solution. The potential is stepped directly to the desired, optimum value and the currents resulting from the redox reaction are detected by the working electrode. Current generated by the reaction (*i.e.*, the current passing through the electrochemical cell over time) is proportional to the concentration of the electroactive species in the sample. Amperometric signal response for back to back additions of the sample usually resembles a “staircase” where it is relatively easy to identify the starting and final current for each addition. One limitation of amperometry is the generation of charging currents (*i.e.*, the current needed to apply the potential to the system) at the beginning of the measurement. What this means is that it takes awhile for the background current to stabilize before quantitative measurements can be made. Amperometric detection was used in the measurement of biomarkers such as CA125 and AFP, as described previously [108,118].

#### 5.2.2. Voltammetric Sensors

Voltammetry is perhaps the most widely used electrochemical method as it is also used for nonanalytical purposes by biochemists, materials scientists, chemical engineers, inorganic, and physical chemists. In addition to quantification of a redox active analyte, voltammetry can be used to: (1) monitor adsorption processes on surfaces; (2) probe electron transfer mechanisms at electrode surfaces that have been chemically modified by nanoparticles or other materials; and (3) perform fundamental studies of oxidation/reduction processes. Voltammetry is a broad term that describes all electroanalytical methods in which the applied potential is scanned over a set potential range at a steady scan rate (mV/s), while the resulting current is measured. Voltammetry is based on the measurement of current that develops in an electrochemical cell under conditions of complete concentration polarization [144]. The resulting current responses from variable potential excitation signals are usually peak(s) or plateau(s) that is/are proportional to the concentration of analyte. Only a minimal consumption of analyte takes place in voltammetry [144]. Voltammetric methods include linear sweep/scan voltammetry, cyclic voltammetry, hydrodynamic voltammetry, differential pulse voltammetry, square-wave voltammetry, ac voltammetry, polarography, and stripping voltammetry. These voltammetric methods generally have wide linear ranges where the signal generated is directly proportional to the analyte concentration, and can be useful for low level quantitation. A two- or three electrode electrochemical detection cell with a potentiostat can be utilized in voltammetry with the three electrode system typically being the more accurate in measuring the current. 

##### Cyclic Voltammetry (CV)

Cyclic voltammetry (CV) is often the first electrochemical experiment performed in the lab after fabrication of a new electrochemical sensor device. CV can be used for mechanistic study of inorganic and organic redox systems. However, CV can also be used for quantitative study of redox active analyte concentrations. For the majority of CV experiments a small, stationary working electrode and the other electrodes are immersed in the electroactive species containing sample solution. Supporting electrolyte, a highly soluble salt such as NaCl or KNO_3_ a nonreactive electrolyte in aqueous samples, is usually added in excess to the test solution to ensure sufficient solution conductivity. The current response of the analyte in the solution is recorded in a response to excitation by a triangular potential wave form [143]. The potential is varied linearly between initial, switching potential (where the scan direction is reversed), and a final potential (usually equal to initial potential applied) *versus* a reference electrode. This excitation cycle can be repeated several times. For an analyte species with reversible redox reaction, the theoretical potential difference between the reduction and oxidation peaks is 59 mV. However, in practice the difference between the reduction and oxidation peaks ranges from 70 to 100 mV. The three-electrode detection cell is the most widely used set up in CV [144].

Furthermore, CV is a type of potentiodynamic electrochemical measurement where the potential is continuously swept (*i.e.*, voltage is varied) between two values linearly as a function of time. Usually in CV, the electrode potential is scanned back and forth in search of redox couples. The rate of change of potential with time is referred to as the scan rate (*v*) in mV/s. The combination of the solvent, electrolyte and specific working electrode material *versus* the reference electrode determines the characteristic range of the detection potentials for each analyte species.

##### Square-Wave Voltammetry (SWV)

SWV is a type of pulse polagrography that is fast and has very high sensitivities [144]. An entire voltammogram may be obtained in less than 10 ms by SWV. The extremely high sensitivity, which is key in immunosensor applications such as cancer biomarker detection, and the availability of commercial instruments capable of SWV have made this electroanalytical method more popular over the past few decades. The excitation signal in a potential *vs.* time plot may look like a “staircase” or a series of increasing pillars and troughs.

#### 5.2.3. Impedimetric Sensors

Incorporation of nanomaterials such as CNTs or gold nanoparticles in electrochemical impedance immunosensors is of great importance due to improved electrical conductivity of sensing interface based on unique conducting properties of the nanoparticles and improved connectivity, easier chemical access to the analyte, and significantly increased electrode surface area [145]. In impedometry, the changes in resistive and capacitive properties of materials in the cell are measured and recorded after perturbation with a small amplitude sinusoidal AC voltage excitation signal of about 2–10 mV [145]. The in-phase current response after a voltage probe is applied dictates the real (resistive) component of the impedance, while the out-of-phase current response determines the fictional (capacitive) component. Impedometry is advantageous because: (1) sensitive experimental measurements may be made because the signal response is indefinitely steady and can be averaged over a long time frame; (2) the resulting response can be treated theoretically by linearized current-potential characteristics; and (3) measurements are over a wide time (10^4^–10^−6^ s) or frequency (10^−4^–10^6^ Hz) range. Since this phenomenon typically works close to equilibrium, detailed knowledge of the current *versus* potential curve over wide ranges of over potential is not required.

Impedance methods are capable of characterizing physicochemical processes of widely differing time constants, sampling electron transfer at high frequency and mass transfer at low frequency [145]. Impedimetric detection is relatively common in electrochemical immunosensors as it can be relatively easy to monitor immunological reactions such as Ab-Ag binding on a transducer surface [2]. The small changes in impedance as a result of Ab-Ag binding are proportional to the concentration of the analyte (the biomarker) in the specimen. The antibodies are often immobilized on the electrode surface or incorporated into a conductive polymer film formed on the surface of a working electrode by electrochemical deposition. The electron transfer resistance at the interface between the electrode and the sample solution changes to a small degree upon the Ag binding event. Being able to directly monitor the formation of an Ab-Ag conjugate, allows a label-free detection system with some important advantages such as: (1) ease of detection; (2) lower cost of analysis; (3) faster measurements; (4) shorter detector response times; and (5) higher signal to noise ratio (which in turn leads to higher sensitivity and lower detection limits) [2]. The main disadvantage of this detection method is that regenerating the sensing surface for a later measurement is typically very time-consuming and may not be reproducible [2].

#### 5.2.4. Conductometric Sensors

Conductometric immunosensors have been well studied by various groups specifically for the detection of tumor markers [119]. A simple conductometric transducer detects changes in the electrical conductivity of the sample solution or a nanomaterial containing medium such as nanowires that result from changes in the composition of the solution/medium during the course of a chemical reaction [2]. These detectors are made of an insulating material that is embedded with graphite, platinum, stainless steel or other metallic pieces that serve as the sensing elements [143]. The metal contacts are placed a fixed distance apart from one another to make contact with a sample solution in which changes in conductivity are determined as the signal. In immunosensors with enzyme labels, the change in conductivity is detected as a result of charged products from enzyme-catalyzed reactions increasing the ionic strength, and ultimately the conductivity of the microenvironment. Biosensors based on conductometric detection can be very simple, sensitive, have low power requirements, low cost, and are compatible with advanced micromachining technologies without requiring a reference electrode [119]. This detection method is also amenable to miniaturization of the biosensor device as well as automated detection strategies. In addition to biomarkers of clinical interest such as AFP, conductometric immunosensors have been developed for the detection of toxins such as aflatoxin B_1_ [146], viruses such as hepatitis B [147], and foodborne pathogens such as enterohemorrhagic *Escherichia coli* O157:H7 and *Salmonella* spp. [148].

## 6. Conclusions

The utilization of various biomarkers that can be detected and quantified using sensitive analytical methods has already become a part of the modern medical diagnosis and treatment of certain diseases. Although many biosensors for biomarkers and other analytes to which nanomaterials have been incorporated have shown significantly improved electrochemical performance and have been described in hundreds of recent publications, most of these biosensor devices still remain in the proof of concept or prototype stages of development. The incorporation of highly conductive nanomaterials into biosensors and immunoassays has lead to increased signal to noise ratios and lower detection limits for biomarkers. Another key reason for amplified sensitivity in these devices and assays that include nanomaterials is the high loading of the biological components, (*i.e.*, enzymes or antibodies). Concerns regarding biocompatibility and toxicity of some of these nanomaterials must be studied further since a great amount of resources, effort and time goes into new immunosensor and assay development, optimization, and characterization annually. Diagnostic applications related to cancer diagnosis are the most common application of these technologies but others exist for psychiatric, cardiovascular, infectious, autoimmune, and neurogenerative diseases. The low concentrations of these biomarkers in relatively complex biological samples such as blood, makes the development of new detection methods quite challenging. Such new detection methods must have: (1) ultralow detection limits; (2) reasonably short assay time; (3) low sample requirement; (4) ease of use; and (5) high-throughput capability. Even with the advent of successful diagnostic tools that have been fully developed, optimized, and validated against existing methods used at clinical settings, the complicated assembly process of the immunosensors and subsequent scaling up to their mass production makes the manufacturing of commercially available devices challenging and expensive. Yet another challenge facing researchers is the storage stability of the biological nanomaterials such as enzymes and antibodies. Ultimately, it is likely to be several years before these devices will fully replace currently used techniques for biomarker detection such as ELISA. However, with constant developments in molecular biology, nanofabrication methods and labeling, nanoinstrumentation, and multiplexing capabilities, rapid, sensitive, selective, and easy-to-use biosensors for measurements in the clinical laboratories will become more commonplace in the near future.
